# A Bioinspired Perceptual Sensor for Spatiotemporal Decoding of Haptic and Pain Stimuli in Prostheses

**DOI:** 10.34133/cbsystems.0679

**Published:** 2026-09-22

**Authors:** Ye Qiu, Shihan Wang, Qiangqiang Qian, Yu Yan, Weisheng Wang, Xiu Jia, Shengwei Fan, Yuan Bao, Ye Tian, Yi Song, Aiping Liu, Liu Wang, Liqiang Zhu, Huaping Wu

**Affiliations:** ^1^ College of Mechanical Engineering, Key Laboratory of Special Purpose Equipment and Advanced Processing Technology, Zhejiang University of Technology, Hangzhou 310023, China.; ^2^ School of Physical Science and Technology, Ningbo University, Ningbo 315211, China.; ^3^ CAS Key Laboratory of Mechanical Behavior and Design of Materials, Department of Modern Mechanics, University of Science and Technology of China, Hefei 230027, China.; ^4^ School of Media and Communication, Shenzhen University, Shenzhen 518000, China.; ^5^ Center for Optoelectronics Materials and Devices, Zhejiang Sci-Tech University, Hangzhou 310018, China.; ^6^ Collaborative Innovation Center of High-end Laser Manufacturing Equipment (National “2011 Plan”), Zhejiang University of Technology, Hangzhou 310023, China.; ^7^ Taizhou Research Institute, Zhejiang University of Technology, Taizhou 318000, China.

## Abstract

Phantom limb pain, a common sequela in lower-limb amputees, impairs their ability to accurately perceive and distinguish between haptic and pain sensations, thereby hindering residual limb function and reducing rehabilitation efficacy. Although existing prosthetic technologies seek to restore sensory function, they often fail to provide real-time localization of haptic stimuli and effectively integrate pain feedback, resulting in an inability to deliver cohesive tactile and nociceptive cues. Here, we present a bioinspired perceptual sensor (BPS) capable of spatiotemporally decoding haptic and pain information by integrating artificial mechanoreceptors with synaptic transistors. High sensing performance and synaptic plasticity of the BPS enable the localization and intensity assessment of haptic stimuli, along with the spatiotemporal integration of pain feedback, facilitating the quantitative evaluation of synergistic haptic and pain perception. We first validated our BPS by integrating it into a humanoid robotic system and demonstrated its ability to distinguish and respond to environment-responsive conditioned and unconditioned reflexes in a closed-loop manner. Then, the sensing platform was incorporated into the prosthetic socket interface for amputees, and its capability to provide accurate, reliable haptic and pain feedback was confirmed, thereby offering crucial insights for optimizing prosthetic fit, limb health, and functional rehabilitation outcomes.

## Introduction

Each year, approximately 5 million individuals worldwide undergo lower-limb amputation, impairing mobility and often resulting in chronic pain perceived as originating from the missing limb, known as phantom limb pain (PLP) [1]. Standard clinical management typically includes prosthetic limb fitting to restore functional mobility, supplemented by neuroleptic and opioid medications to alleviate PLP [2]. Despite these interventions, damage to mechanical and nociceptive sensory receptors in the residual limb often leads to impaired perception of haptic and painful stimuli, inevitably resulting in severe complications [3–5]. For example, high pressures distributed at the interface between the prosthetic socket and the residual limb may cause ulcers due to excessive load on the skin and underlying bony structure [6]. Thus, restoring accurate and timely haptic and pain feedback at the prosthesis and residual limb interface, along with adaptive adjustments of prosthetic function that promote natural motor control, is crucial for improving prosthetic integration and functional outcomes in closed-loop perception and action rehabilitation [7–10]. Although pressure sensing and load monitoring at prosthetic interfaces have been explored [11,12], existing systems still rarely provide synergistic haptic localization and pain-related feedback comparable to human somatosensory perception, underscoring the need for bioinspired sensing systems that can deliver precise tactile information and timely protective pain alerts for prosthetic fit assessment and rehabilitation feedback [13–18].

Recognizing the critical role of somatosensory feedback and current prosthetic limitations, substantial research efforts have been devoted to artificial sensing technologies for sensory restoration in amputees [19–26]. Recently developed flexible tactile sensors utilize advanced materials and structural innovations to convert mechanical signals into electrical responses, and typically distinguish between normal tactile sensations and pain using predefined external thresholds [27–33]. Despite improvements in sensitivity, stretchability, and stability, these devices still lack the intrinsic signal processing and memory capabilities characteristic of biological neural systems, which rely on synaptic neurotransmitter release to mediate plasticity (strengthening or weakening synaptic connections), learning, and memory formation. To bridge this gap, recent strategies have introduced synapse-inspired neuromorphic elements—such as memristors or transistors—into pressure-sensing systems, integrating sensing, signal processing, and memory functions, and thus facilitating a transition from basic sensory detection to perception and cognition [34–42]. Various artificial neuromorphic sensory systems, employing combinations of piezoelectric, triboelectric, or pyroelectric sensors coupled with field-effect or electrolyte-gated transistors, have been explored to decode spatial and temporal characteristics of pain stimuli. Such integration leverages computational advantages, compatibility, and synaptic plasticity provided by transistor-based designs [43–53]. Nevertheless, existing implementations still struggle to simultaneously achieve precise real-time localization required for haptic perception and the spatiotemporal integration essential for pain sensing, presenting an important barrier to practical applications.

**Fig. 1.**
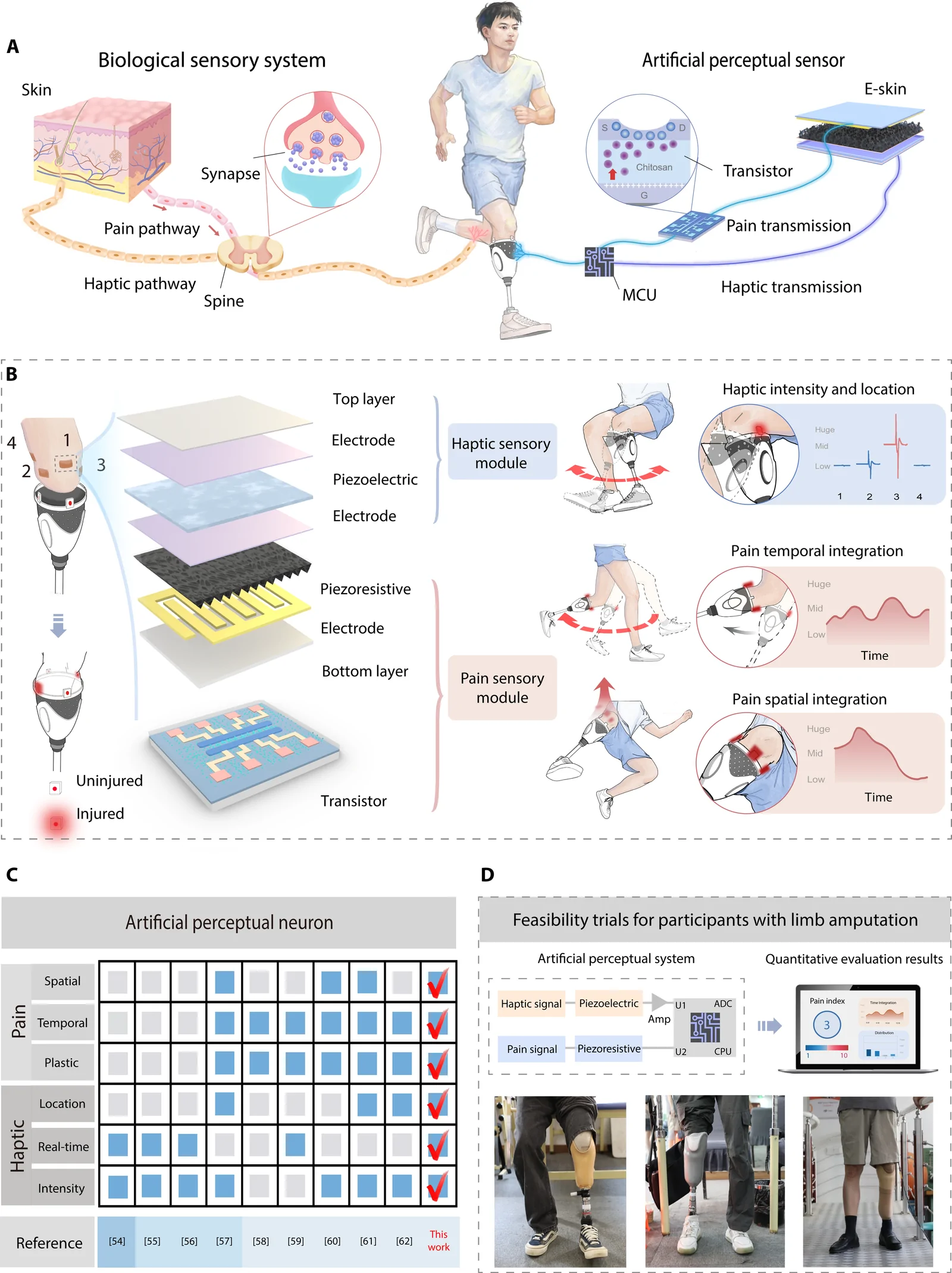
A BPS for spatiotemporal decoding of haptic and pain stimuli in prostheses. (A) Schematic comparison of the BPS architecture and its tactile sensing system with the biological haptic and pain sensory system in human skin. (B) Detailed structure of BPS comprising a piezoelectric-based haptic sensing module for real-time localization and intensity detection, and a piezoresistive-based pain sensing module integrated with electrolyte-gated transistors to achieve pain-related temporal integration and synaptic plasticity. (C) Comparison of the sensory functionality between our BPS and previously reported sensing systems, highlighting the more comprehensive and robust sensory performance of our design. (D) Implementation schematic and feasibility trials showing the BPS integrated into prosthetic limbs, providing accurate quantitative evaluation of haptic sensations and pain stimuli, thus aiding in prosthesis fitting, limb health monitoring, and rehabilitation for individuals with limb amputation.

In this work, we developed a BPS that closely mimics biological mechanoreceptors and nociceptors, enabling simultaneous decoding of spatiotemporal haptic and pain stimuli for lower-limb amputees (Fig. 1A). Our integrated sensing platform comprises 2 synergistically operating modules: a piezoelectric-based haptic sensing module that provides real-time spatial localization and intensity detection, and a piezoresistive-based pain sensing module incorporating electrolyte-gated transistors for spatiotemporal integration and neuroplasticity in response to pain stimuli (Fig. 1B). Compared to existing artificial sensory systems [54–62], our BPS offers enhanced perceptual fidelity and comprehensive sensory feedback, more accurately emulating human tactile and pain sensations (Fig. 1C). We first validated our BPS by integrating it into a humanoid robotic system and demonstrated its ability to distinguish and respond to environment-responsive conditioned and unconditioned reflexes in a closed-loop manner. Then, the sensing platform was incorporated into the prosthetic socket interface for amputees (Fig. 1D) and its capability to provide accurate, reliable haptic and pain feedback was confirmed, thereby offering crucial insights for optimizing prosthetic fit, limb health, and functional rehabilitation outcomes.

## Materials and Methods

### Preparation of laser-induced graphene (LIG) sensors

A CO_2_ infrared laser was used to scan a 50-μm polyimide (PI) film, resulting in the creation of LIG with porous structures (Fig. S1). A 10:1 uncured polydimethylsiloxane (PDMS, Sylgard 184, Dow Corning) solution was then used to cover the LIG surface, followed by vacuum treatment to eliminate bubbles and ensure full infiltration. The coated LIG material was placed in a 70 °C environment for 2 h to cure. After curing, the LIG/PDMS flexible film was separated from the PI substrate, transferring the LIG onto the PDMS. Finally, the LIG/PDMS was encapsulated with interdigital electrodes, coated with 150 nm of Cu and 10 nm of Au, resulting in a piezoresistive flexible sensor (Fig. S2).

### Preparation of piezoelectric sensors

The poly(vinylidene fluoride-trifluoroethylene) P(VDF-TrFE) powder (Piezotech) was combined with *N*,*N*-dimethylformamide in a 70:30 molar ratio. After stirring the mixture at room temperature for 2 h, a uniform solution was obtained. This solution was cast into a petri dish and annealed at 120 °C for 2 h to improve crystallinity, resulting in the formation of a P(VDF-TrFE) film. Once the film was peeled from the petri dish, it was polarized in a silicone oil bath under an electric field of 70 MV/m for 30 min. This polarization process enhanced the film’s piezoelectric properties, achieving a piezoelectric strain constant (*d*_33_) value of 25 pC/N along the *z*-direction, which was measured using a quasistatic *d*_33_ tester (ZJ-6A from the Chinese Academy of Sciences) at room temperature. The piezoelectric film, cut to 7 mm × 8 mm, has deoxidized copper wires bonded to both sides with silver paste, allowed to dry, and was then encapsulated with PI film before being heated for 30 min to prepare a piezoelectric sensor (Fig. S3).

### Chitosan membrane synthesis and characterization

At room temperature, 1.25 g of chitosan powder, 60 g of deionized water, and 1.35 g of acetic acid were combined in a beaker, mixed thoroughly, and allowed to put in a dark place for 24 h. The chitosan solution was then applied to indium tin oxide (ITO)-coated conductive glass, which was dried in an oven at 35 and 45 °C for 6 h each. Subsequently, ITO electrodes were deposited on the chitosan film using an RF magnetron sputtering system, and the impedance of the film was measured to obtain its equivalent circuit diagram (Fig. S4).

### Preparation of transistors

The fabrication of chitosan thin-film transistors uses the same materials and equipment as the chitosan electrolyte films, with the only difference being the use of a shadow mask for depositing ITO source/drain electrodes and channels during sputtering. By controlling the distance between the mask and the chitosan film, a self-diffraction effect was formed, resulting in ITO source/drain electrodes and channels with a central square aperture size of 150 μm × 1,000 μm and a spacing of 80 μm between the apertures (Fig. S5).

### Integration of the BPS system

The BPS system was assembled by coupling the 2 × 2 bimodal flexible sensing arrays with a chitosan-gated ITO synaptic transistor and peripheral signal-conditioning circuits. In this architecture, the P(VDF-TrFE) piezoelectric and LIG/PDMS piezoresistive units acted as front-end dynamic and static mechanosensory inputs, whereas the synaptic transistor served as the neuromorphic core for signal integration and plastic modulation. The sensing outputs were delivered to the transistor and acquisition circuit through wired interconnections, with voltage-divider circuits, charge amplifiers, and microcontroller-based modules used for signal conditioning and judgment. This integrated configuration enabled parallel tactile sensing and pain-related neuromorphic processing under mechanical stimulation.

### Device and system characterization

Compression tests of the sensing system were conducted using a mechanical testing system (Legend2345, Instron). The electrical output of the transistor and piezoresistive sensor were measured through a semiconductor parameter analyzer (4200-SCS, Keithley). The voltage output of the piezoelectric sensor was recorded using a piezoelectric data acquisition system integrated with a high-precision network data analyzer (KSI-8904N), a power amplifier (KSI-758PA100), and a charge amplifier (KSI-608A100).

## Results

### Biomimetic design and working principle of the BPS

**Fig. 2.**
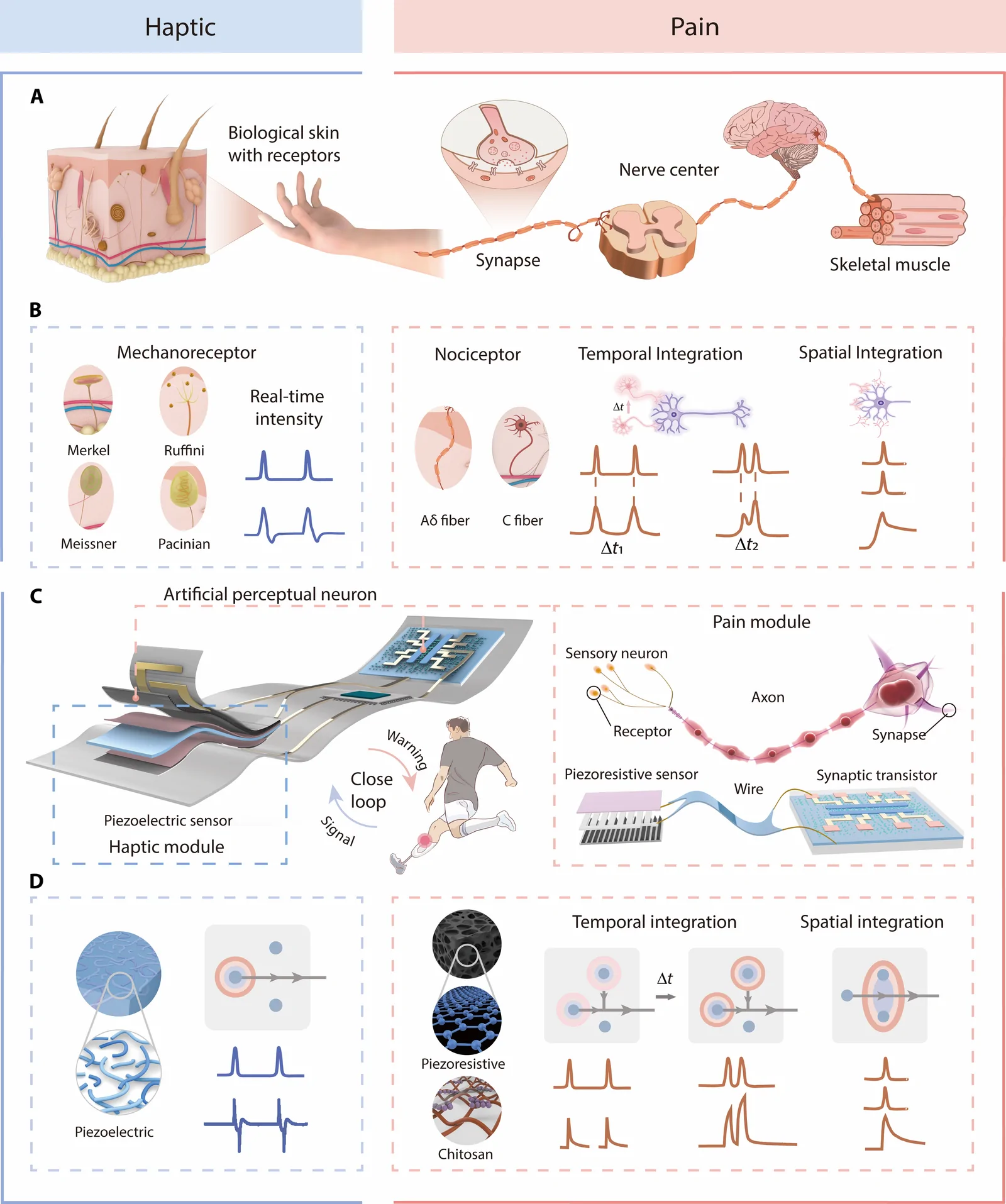
Biomimetic design and working principle of the BPS. (A) Biological basis of human somatosensory perception involves the detection of tactile and noxious stimuli by mechanoreceptors and nociceptors in the skin, transduction into action potentials that are transmitted via distinct ascending pathways to the thalamus and processed in the primary somatosensory cortex for conscious perception, while integration in additional cortical and subcortical areas contributes to affective evaluation and motor response coordination. (B) Expanded view depicting biological mechanoreceptors responsible for real-time intensity sensing, and nociceptors involved in temporal and spatial integration of pain signals. (C) Structural configuration and working principle of the BPS system. The haptic module employs piezoelectric sensors for real-time localization and intensity sensing, while the pain module utilizes piezoresistive sensors integrated with electrolyte-gated synaptic transistors to achieve temporal integration and spatial mapping of pain stimuli, thereby providing simultaneous and synergistic sensory feedback for amputees in a closed-loop control manner. (D) Expanded views highlighting the piezoelectric-based haptic module, capable of detecting stimulus intensity in real time, and the piezoresistive-based pain module, demonstrating its capability to integrate temporal and spatial signals, mimicking biological sensory neurons for enhanced prosthetic sensory feedback.

To design a BPS, we first explored the neural mechanisms underlying human skin’s haptic and pain perception. Physiologically, tactile and noxious stimuli sensed by cutaneous receptors elicit action potentials that are transmitted via afferent neurons into the spinal cord. Tactile signals are relayed through the dorsal column–medial lemniscal pathway, while pain signals are transmitted via the spinothalamic tract [63,64]. Both pathways ultimately reach the thalamus and then the primary somatosensory cortex [65], where they undergo further processing before being integrated in higher-order association areas for perception and decision-making (Fig. 2A). Importantly, tactile signals enable real-time encoding of stimulus intensity and location, underpinning fine discrimination for environmental interaction, whereas nociceptive signals rely on spatiotemporal integration to detect threats and execute protective behavior [66] (Fig. 2B). Specifically, tactile information from low-threshold mechanoreceptors (e.g., Merkel, Ruffini, Meissner, and Pacinian corpuscles) is primarily conveyed by fast-conducting, thickly myelinated Aβ fibers. First-order neurons in the dorsal root ganglia ascend ipsilaterally via the dorsal columns to synapse onto second-order neurons in the gracile and cuneate nuclei of the medulla, whose axons decussate and form the medial lemniscus to ascend to third-order neurons in the ventral posterior lateral nucleus of the thalamus, which then relay the information to the primary somatosensory cortex for real-time tactile intensity perception and localization [67,68]. Meanwhile, pain signals originate from nociceptors and are primarily transmitted by unmyelinated C fibers and thinly myelinated Aδ fibers [69,70], traveling through the dorsal root ganglia into the posterior horn of the spinal cord, ascending via the spinothalamic tract to the ventral posterolateral nucleus of the thalamus, and subsequently relayed to the somatosensory cortex for the processing and perception of pain. Notably, the encoding of pain stimuli relies on 2 key mechanisms: temporal integration (summation of repeated noxious inputs over time) and spatial integration (convergence of signals from adjacent nociceptors across a region).

Inspired by this biological sensory function, we developed a BPS composed of integrated haptic and pain sensing elements designed to decode spatiotemporal stimuli (Fig. 2C). The haptic module employs a flexible piezoelectric sandwich structure [Au/P(VDF-TrFE)/Au], which generates immediate voltage signals in response to mechanical pressure through dipole reorientation and charge displacement, thereby enabling real-time sensing of the intensity and location of tactile stimuli (Fig. 2D). Meanwhile, the pain sensing module features a piezoresistive sensor integrated with a synapse-inspired electrolyte-gated transistor, capturing both spatial and temporal information to closely mimic the integrated spatiotemporal behavior of nociceptive pathways. Specifically, this pain module incorporates LIG transferred onto PDMS as the sensing layer, with gold interdigital electrodes deposited on a PI substrate. Upon pressure, deformation of the porous LIG network alters its conductive pathways, leading to measurable resistance variations (Fig. S6A). Collectively, the artificial sensory neuron system is designed to emulate the overall sensory pathway of pressure perception, rather than a single peripheral receptor. The haptic module primarily mimics peripheral mechanoreceptors for rapid, real-time pressure detection while the pain module incorporates synaptic-like processing to capture central pathway features such as spatiotemporal integration, with the haptic pathway simplified to focus on protective reflexes and emphasize its essential functional role within the system’s inherent plasticity. Together, these biomimetic designs facilitate adaptive, closed-loop sensory feedback for prosthetic limb control.

### Performance of haptic and pain sensory receptors

**Fig. 3.**
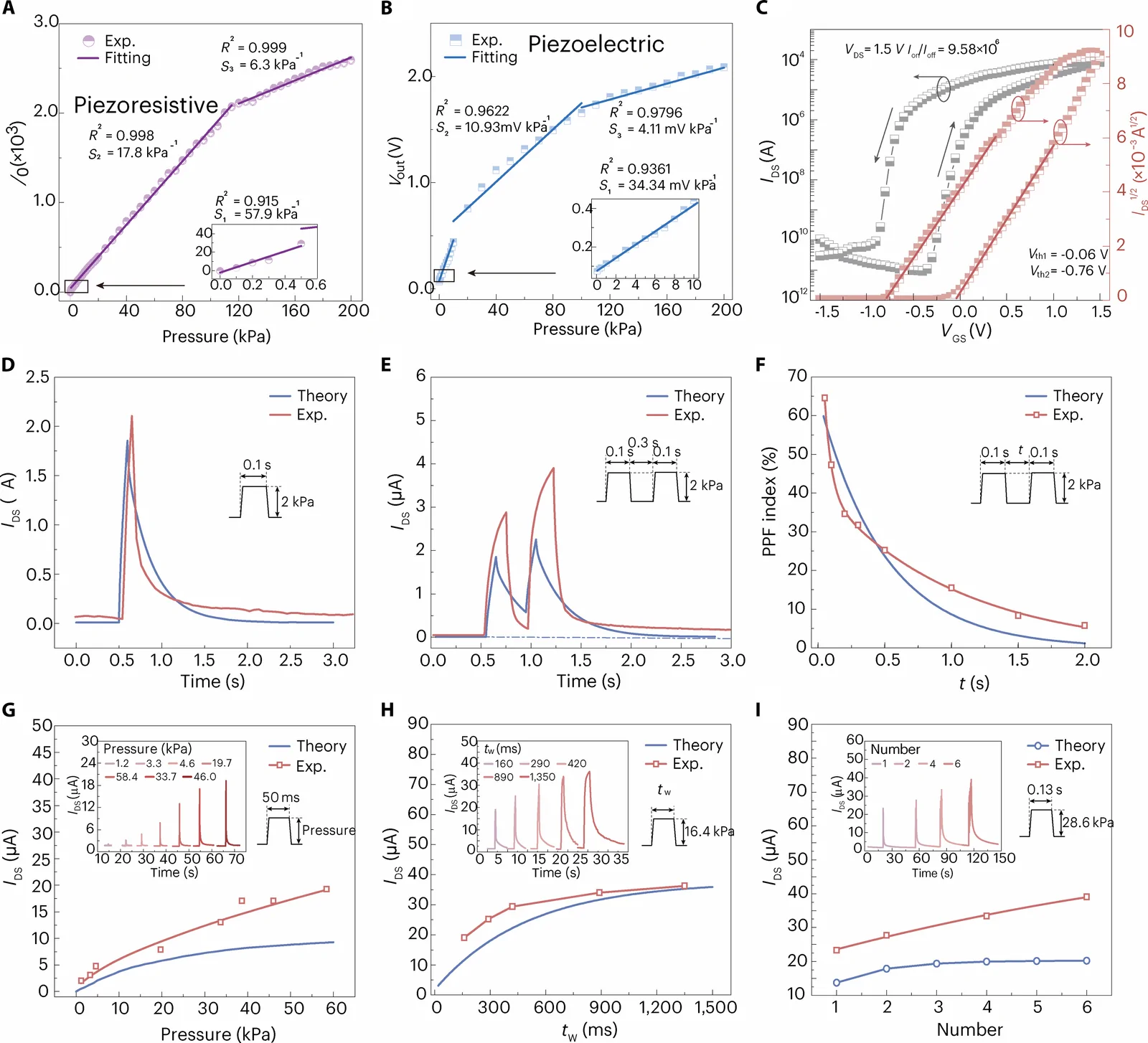
Biomimetic design and working principle of the BPS. The sensitivity of bimodal sensors based on (A) piezoresistive and (B) piezoelectric mechanisms varies when the applied forces range from 0 to 200 kPa. (C) The transfer characteristics of the transistor under the operating voltage of −1.5 to 1.5 V. A comparison of typical (D) EPSC, (E) PPF response, and (F) PPF index of the pain sensing element between the theoretical predictions and experimental demonstrations. A comparison of the (G) pressure strength, (H) duration time, and (I) number-dependent EPSC responses of the pain sensing element between theoretical predictions and experimental demonstrations. Insets show the real-time measured EPSC responses.

To evaluate the sensing performance of the BPS, we first characterized the LIG-based piezoresistive pain sensor by systematically measuring the relationship between electrical current variations and applied pressure. The piezoresistive sensor exhibits high sensitivity (57.9 kPa^−1^) in the low-pressure range (100 to 500 Pa), maintains robust sensitivity (17.8 kPa^−1^) up to 120 kPa, and still shows a sensitivity of 6.3 kPa^−1^ in the 100 to 200 kPa range (Fig. 3A). The real-time current changes of the piezoresistive sensor show a substantial increase toward different levels of pressure (Fig. S6B and E). Real-time tests reveal that the sensor reliably detects varying pressure magnitudes with rapid response and recovery times of 31 and 43 ms, respectively (Fig. S6B). The real-time resistance decreases as the applied pressure increases, yielding an obvious resistance change rate (Fig. S6C). The linearity of all typical current–voltage curves under various pressure reveals that the favorable ohmic contacts formed between the piezoresistive film and electrodes (Fig. S6D). The stability and robustness were investigated by periodically applying 2,000 cyclic pressure at 4 kPa, and the sensor still works well without any notable performance degradation (Fig. S6F).

To further characterize the sensing performance of the piezoelectric sensor, voltage outputs of the piezoelectric sensor after poling under different compressive pressure were obtained (Fig. S7A and B). The sensitivity is 34.34 mV/kPa in the low-pressure range (0.1 to 20 kPa), 10.93 mV/kPa under 20 to 100 kPa, and 4.11 mV/kPa in the 100 to 200 kPa range, respectively (Fig. 3B). The centers of both positive and negative charge carriers are not fully separated, leading to a high sensitivity for a smaller applied pressure. Then, the increase in the applied pressure results in a further separation between the centers of positive and negative charge carriers before reaching a maximum value (i.e., the maximum number of dipoles that could orient in one direction), corresponding to the upper limit in the pressure range. The sensor also illustrates ultrafast response (5 ms) and recovery time (4 ms) to both loading and unloading measurements (Fig. S7C), implying an excellent sensing performance in detecting dynamic pressure. The voltage response exhibits excellent repeatability with no signal drift or fluctuation and negligible hysteresis during the 2,000 cyclic tests (Fig. S7D). Comparative analysis reveals that our sensor demonstrates a broad sensing range with high sensitivity, outperforming the tactile sensors reported in the literature (Figs. S8 and S9 and Tables S1 and S2).

### Characterization of pain sensory module

For emulating biological synaptic behaviors in an artificial tactile perceptual neuron, a chitosan-gated ITO neuromorphic transistor array with a device density of 100 transistors per cm^2^ was designed and fabricated (Fig. S10A). The prepared chitosan film demonstrates excellent electrical insulation and proton conductivity, making it a promising candidate material for transistor devices (Fig. S10B to D). The operating mechanisms of the transistor involve the protonic electronic double layer at the chitosan/ITO channel interface, as well as protonic electrochemical doping and de-doping in the ITO channel (Fig. S11). The transfer curve of chitosan-gated synaptic transistor exhibits a noticeable hysteresis window (~0.6 V), high on/off current ratios (~9.6 × 10^6^) and low subthreshold swing (~81.2 mV/decade) (Fig. 3C), with the wide hysteresis reflecting strong nonvolatile memory from ion migration and trap dynamics. This phenomenon occurs during voltage forward scanning, where protons accumulate at the chitosan/ITO interface, enhancing conductivity through electrostatic coupling, while their delayed return in reverse scanning creates a hysteresis window. The chitosan-based electrolyte demonstrates excellent insulating properties with a low leakage current of less than 40 nA for the sweeping test ranging from −1.5 to +1.5 V (Fig. S12A). In addition, the transfer curves of the transistor were measured 5 times, confirming its electrical stability (Fig. S12B). The device clearly exhibits a strong ohmic contact and has typical electrical characteristics of an N-type transistor, as evident from the output curve (Fig. S12C). Besides, we have developed an analytical model for quantitatively describing the steady-state response of transistors (see text S1-1.1 for details). Our analytical model’s predictions closely match the experimental results, including transfer and output characteristics (Fig. S12C and D).

Fundamental transient behaviors of synaptic plasticity encompass short-term plasticity (STP) and long-term plasticity (LTP) acting as the underlying mechanisms of the cellular biology for brain learning and memory. In the pain pathway, synaptic plasticity is closely associated with nociceptive intensity coding, temporal summation, and sensitization. Therefore, the transient excitatory post-synaptic current (EPSC)/paired-pulse facilitation (PPF) responses and sustained LTP-like conductance enhancement in the BPS system can be used to emulate acute pain responses and repeated stimulation-induced pain sensitization, respectively. The theoretical analysis (see text S1-1.2 for details) and experimental observation on the transient behaviors of the transistor consistently revealed electric-induced response (Fig. S13). Primary forms of STP are EPSC and PPF (a short-term enhancement of synaptic responses to 2 closely spaced stimuli), both of which play important roles in decoding temporal information in sensory nervous systems. For instance, in EPSC response, information from the presynaptic membrane will transiently induce the postsynaptic membrane to fire an action potential (Fig. S14A), while in PPF responses, 2 consecutive presynaptic spikes can activate enhanced and facilitated EPSCs (Fig. S14B). The enhancement is commonly evaluated by a PPF index from the equation (*A*_2_ − *A*_1_)/*A*_1_, where *A*_1_ and *A*_2_ are the peak values of these 2 pulses. As the interval increases from 39 ms to 1.115 s, the index decreases exponentially from 57% to 4.3%. Then, the data were fitted with the equation of double-exponential decay function: PPF index = *C*_1_ · exp(−Δ*t*/*τ*_1_) + *C*_2_ · exp(−Δ*t*/*τ*_2_), where *C*_1_ and *C*_2_ are initial values of the rapid and slow phases, respectively. The rapid phase time (*τ*_1_) was ~10.2 ms, and the slow phase time (*τ*_2_) was ~292.1 ms (Fig. S14C). Then, its processing behaviors were examined by applying pulse voltages with a series of magnitudes, durations, frequencies, and numbers to the transistor and collecting the postsynaptic currents of the system (Fig. S14D to F). When the applied gate voltage exceeds approximately 4 V, the operational mode of the synaptic transistor shifts from STP to LTP, causing drain current to exhibit sustained conductance enhancement due to electrochemical doping. Increasing the frequency, duration, amplitude, and number of voltage pulses to accumulate more charge carriers enhances the postsynaptic current and prolongs the time to the resting potential, demonstrating the feasibility of chitosan-gated neuromorphic transistors in modulating synaptic weights (Fig. S15). Through further modulation of voltage magnitude and duration, the synaptic transistor successfully replicates the action potential responses associated with biological sensitization and desensitization states (Fig. S16).

Considering the proposed piezoresistive sensors with perceptual abilities akin to biological receptors and neuromorphic devices that mimic synaptic behaviors, the chitosan-gated ITO synaptic transistor was integrated with the piezoresistive sensor to construct an artificial tactile perceptual neuron. To obtain an in-depth understanding of the STP mechanism and account for the applied pressures, an electromechanical model of the proposed device was established based on its equivalent circuit diagram (details shown in the text S2). A theoretical EPSC response was triggered on the ITO neuromorphic transistor with a constant *V*_DS_ of 0.5 V, concurrent with loading a pressure on the piezoresistive sensor with a constant bias *V*_GS_ of 1.5 V (Fig. 3D, blue line). In terms of PPF, applying 2 successive loading pressures with a time interval of 0.3 s to the device enables the second pulse to enhance and facilitate EPSCs (Fig. 3E, blue line). Corresponding loading and electrical tests were conducted on the fabricated transistor, and the obtained EPSC (Fig. 3D, red line) and PPF (Fig. 3E, red line) emulation results are in good agreement with the theoretical prediction in biological systems. This is attributed to the time interval being shorter than the relaxation duration of the protons, hence impeding the diffusion of some protons back to their equilibrium location after being activated by the initial pulse, before the arrival of the second pulse. The theoretical and experimental facilitation ratio both decreases with the increase of the time interval (Δ*t*) between the pair of stimuli (Fig. 3F), which is similar to the behavior of biological tactile sensory neuron, and conveys temporal messages of the applied pressures. EPSCs triggered by external pressures with different intensities, frequencies, and amounts were simulated, thereby modulating the memory behavior of artificial synapse (Fig. 3G to I). The pain perceptual behaviors of the artificial tactile perceptual neuron closely resemble biological EPSCs, which are influenced by various presynaptic spikes and neuron activities, and are crucial for collecting and refining complex dynamic sensory data.

### Real-time synergistic haptic and pain perception

**Fig. 4.**
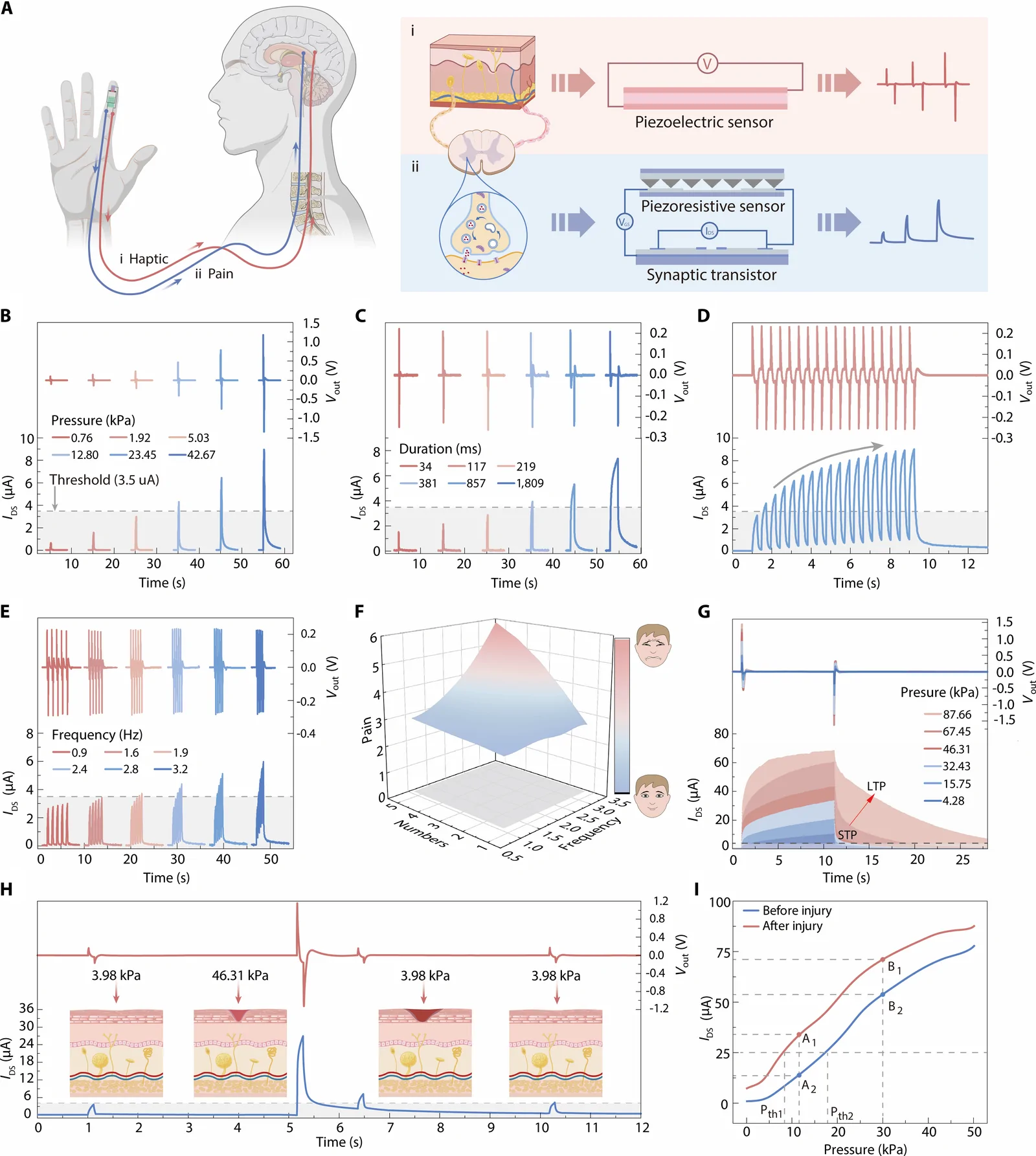
Real-time synergistic haptic and pain perception enabled by integrated sensory feedback. (A) Schematic diagram of the combination of haptic and pain sensory feedback in biological skin. Panel (i) shows a schematic of the device structure and electric circuit for haptic sensing response. Panel (ii) is a schematic illustration of showing the pain sensing response with the integrated pressure sensor and synaptic transistor. Measured voltage and current in haptic and pain sensing elements as the applied pressure varies the (B) magnitude, (C) duration, (D) number, and (E) frequency. (F) Color mapping of pain intensity changes governed by pressure numbers and frequency of the haptic and pain sensing elements. (G) The outputs of haptic and pain sensing elements under the sustained pressure (duration time: 10 s) loading. (H) The real response of pressure magnitude applied and released time in haptic sensing elements, and the simulated pain sensitization and desensitization process in the pain sensing elements. (I) Relationship between output currents and applied pressures before and after injury in pain sensing element.

With all essential components in the e-skin system reproducing the biological perception and processing behavior, we next addressed the need to synergistically perceive haptic and pain through a neuromorphic interface that produces receptor-like spiking neural activity. A BPS was designed to further exploit the multifunctional perceptions and primarily comprises a haptic sensory pathway made up of the piezoelectric layer, along with a pain sensory pathway composed of the piezoresistive layer and synaptic transistor (Fig. 4A). To assess the sensing capabilities of the BPS system, we examined its electrical outputs when exposed to various forms of pressure, including variations in intensity, duration, number, and frequency. The output voltage of the haptic sensing element increases proportionally with the load, enabling precise differentiation between harmless and harmful stimuli. Upon reaching a load intensity of 12.80 kPa, the EPSC of the pain sensing element surpasses the threshold of 3.5 μA, which was chosen based on the observable onset of current relaxation phenomenon, signifying the emergence of pain and the transition from a nonmemory state to STP, with higher EPSC values reflecting more intense pain sensations (Fig. 4B). Meanwhile, the haptic sensing element has outstanding dynamic perception by accurately detecting stimulus application and withdrawal times, as evidenced by its consistent output signal throughout pressure of varying durations. In contrast, the signal strength from the pain sensing element increases as the pressure duration increases, indicating that the intensity of pain is related to the duration of external stimuli (Fig. 4C). When the device was subjected to different numbers and frequencies of pressure (Fig. 4D and E), the output signal strength of the haptic sensing element remains constant regardless of the parameter of pressure, whereas the response of the pain sensing element becomes stronger as the pressure number and frequency continue. Assessing the synergistic effect of the artificial haptic neuron system reveals that the pain perceived can be accumulated through measured features (i.e., pressure intensity, frequency, and number of pressure), while the haptic sensing depends only on real-time pressure intensity (Fig. 4F).

To explore a deeper understanding of its haptic and pain sensing mechanism, pressures of varying magnitudes were applied to the device, with the duration of each pressure kept consistent for a period of 10 s (Fig. 4G). It was found that the haptic element enables real-time measurement of pressure intensity, and applied and removed timings. The pain element exhibits a no-adaptation feature of biological nociceptors, as its response exceeds the pain threshold and continuously increases under constant pressure, with shorter durations to reach the threshold but longer recovery times at higher-amplitude pulses, reflecting a shift in pain perception from short-term to long-term memory. Furthermore, in biological systems, pain perception involves key protective features such as sensitization and desensitization, in which sensitization enhances nociceptor sensitivity to noxious stimuli to safeguard injured areas, and desensitization restores normal pain thresholds once the stimulus subsides. To emulate these processes in our artificial perceptual neuron, we applied external pulse stimuli with varying intensities and intervals (Fig. 4H). Specifically, a mild stimulus (3.98 kPa, 0.2 s) was insufficient to evoke a noticeable pain response, followed by a strong stimulus (46.31 kPa, 0.2 s) that triggered intense pain and mimicked an injury state. When the same mild stimulus was applied shortly afterward with a short interval (3.98 kPa, 1.4 s), the sensor exhibited an enhanced response, corresponding to pain sensitization after injury. In contrast, when the mild stimulus was applied with a longer interval (3.98 kPa, 4 s), the response returned to the normal level, reflecting the desensitization process. It can also be characterized in pain element by the reduced pain threshold value (i.e., *P*_th1_ < *P*_th2_), referring to a higher level of pain response to a normally painful stimulus (*B*_1_ > *B*_2_) and a pain feeling resulting from a normally nonpainful stimulus (*A*_1_ > *A*_2_), respectively (Fig. 4I). These results demonstrate that our artificial perceptual neuron can faithfully emulate pain sensitization and desensitization characteristics while maintaining accurate haptic perception, thus providing potential solutions to avoid secondary injuries.

### Closed-loop response of human–robot interaction

**Fig. 5.**
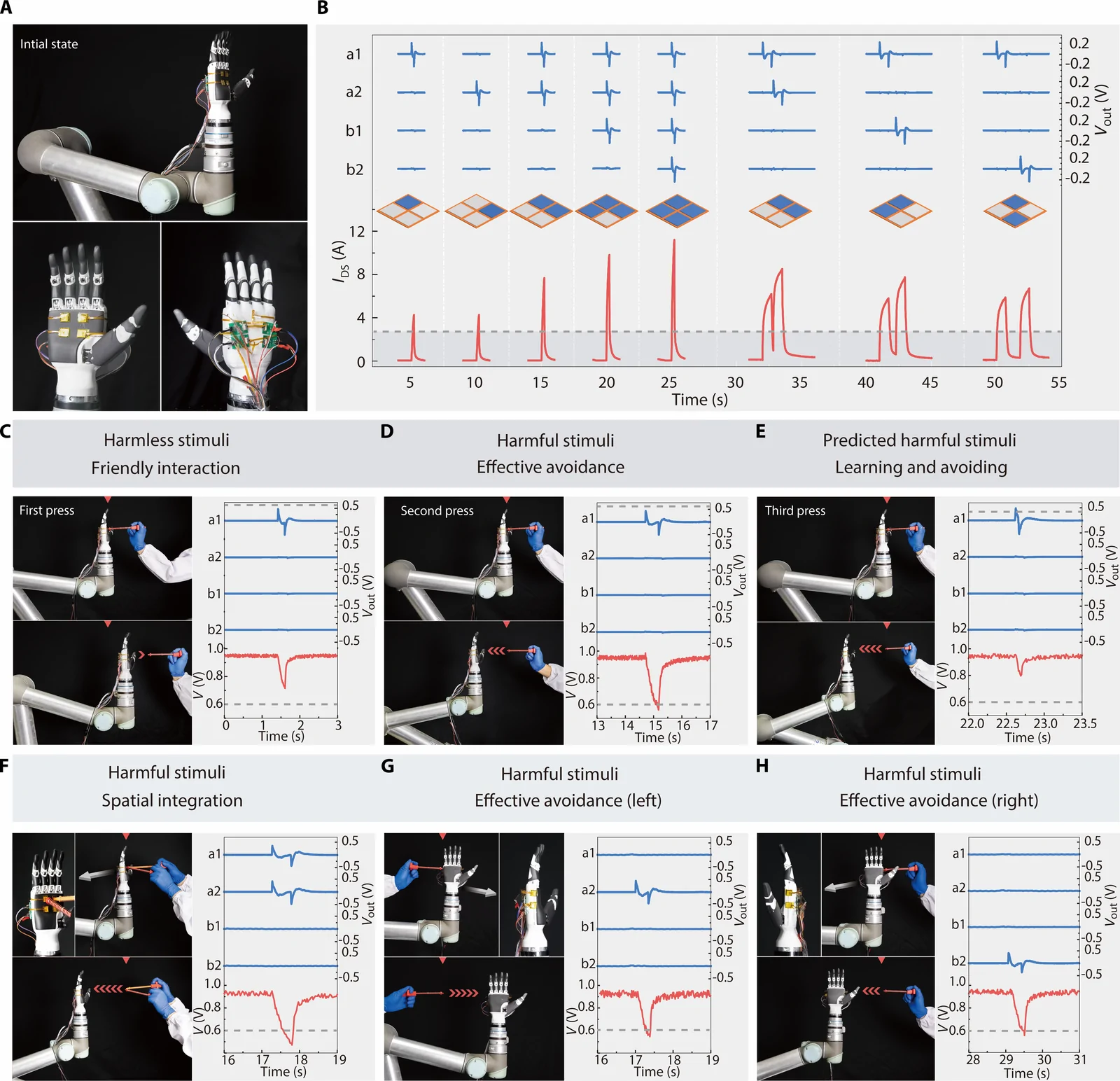
Validation of haptic and pain perception in human–robot interaction through a closed-loop system. (A) The optical image of the artificial perceptual system integrated into the robot hand as a safe communication interface to facilitate friendly human–robot interaction. (B) Spatiotemporal decoding magnitude and location of haptic and pain information through the artificial perceptual system. (C) The robot hand detects the harmless stimuli and triggers a friendly human–robot interaction. (D) The robot hand senses the harmful stimuli and performs a forward flip action to dodge the danger. (E) The robot hand adjusts the threshold to achieve active protection in advance through learning. (F) Real-time pain integration, (G) left and (H) right location of the robot hand assisted by the artificial perceptual system.

The ability to simultaneously perceive pain and haptic sensations through an artificial device enhances the control of tasks performed by robots, particularly those requiring advanced dexterity. A 2 × 2 sensing array was designed to further explore our device’s merits in perception functionalities (Fig. 5A and Fig. S17), thereby enabling robots to acquire a synergistic understanding of pain and haptic information. In this configuration, the synaptic transistor introduces temporal memory and activity-dependent modulation into the pain perception pathway, allowing the history of mechanical stimulation to modulate subsequent pain-related outputs beyond direct piezoresistive signal readout. The EPSC output of the pain perception element enhances as the same force is concurrently applied to the increased number of spatial sensing units, reflecting spatial integration of pain stimuli in a manner consistent with the physiological characteristics of the nociceptive system. Then, 2 consecutive damage stimulations, incorporating 3 distinct directions of movement, are applied to the sensor, effectively simulating the temporal integration characteristic of biological pain perception in the pain sensing element (Fig. 5B). In contrast, the haptic sensing element with 4 channels has the capability to accurately identify the precise location, timing, and magnitude of applied damage stimuli. Thus, by combining spatiotemporal integration effects in pain sensing elements, along with the accurate spatiotemporal localization in haptic sensing elements, an artificial device with synergistic perception of pain and haptic capabilities in temporal and spatial dimensions can be constructed.

We further designed an artificial neuromuscular system to replicate the functions of haptic sensing and injury pain warning, enabling it for friendly human–robot interaction. To adapt our artificial device to real scenarios, the developed closed-loop interactive system facilitates the integration of sensing elements, power supply, microcontroller module, data acquisition module, and robotic manipulator (Fig. S18A). Among them, the microcontroller analyzes the real-time haptic and pain signals and transmits them to the robot hand control component to execute actions according to a threshold-based strategy (Fig. S18B and C). An impulse stimulus (intensity: 10 kPa, duration: 0.21 s) was first applied to the robotic manipulator, and the recorded perceptual information indicated that it was harmless, as it did not reach the predefined pain threshold for intensity or duration, thereby enabling the manipulator to interact with external stimuli in a safe and appropriate manner (Fig. 5C). Then, under a prolonged (0.49 s) stimulus of the same intensity, the pain threshold was exceeded due to temporal integration, causing the robot to immediately recognize the signal as painful and initiate an avoidance action, analogous to a human withdrawal reflex in response to damaging mechanical stimuli (Fig. 5D). Moreover, after this painful experience, the system adapted by lowering the predictive threshold, which allowed the robotic manipulator to recognize the potential risk earlier and respond with a faster withdrawal to subsequent similar stimuli (at a stimulus duration of only 0.06 s, Fig. 5E). This result demonstrates a form of learning-based sensitivity enhancement, analogous to conditioned reflexes or motor memory in biological systems, allowing the robot to improve its self-protection through prior experience.

The strategy of fusing haptic and pain feedbacks can enable robotic motion control, including interacting with harmless stimuli and quickly avoiding harmful stimuli. To implement more complex tasks of self-awareness and self-protection for the robotic manipulator, stimuli from multiple regions and directions are required. When identical stimuli were applied to the 2 sensing elements simultaneously, the artificial system identified the current load as an injury stimulus and controlled the robotic arm to move backward (Fig. 5F). The robotic arm exhibits a substantial increase in both avoidance distance and speed due to the simultaneous stimulation, effectively mimicking the spatial integration of human pain perception and the principle that stronger pain elicits a more pronounced response. Afterwards, the identical stimulus was applied to both the left and right sides of the robotic arm, respectively. The artificial system detects the injury stimulus and its position through the pain and haptic sensing elements, promptly controlling the robotic arm to move in the direction of the stimulus to simulate pressure-assisted pain localization (Fig. 5G and H). These results demonstrate that the robotics with synergistic pain and haptic perception capability can effectively respond to various stimuli.

### Feasibility trials with participants with transtibial amputation

**Fig. 6.**
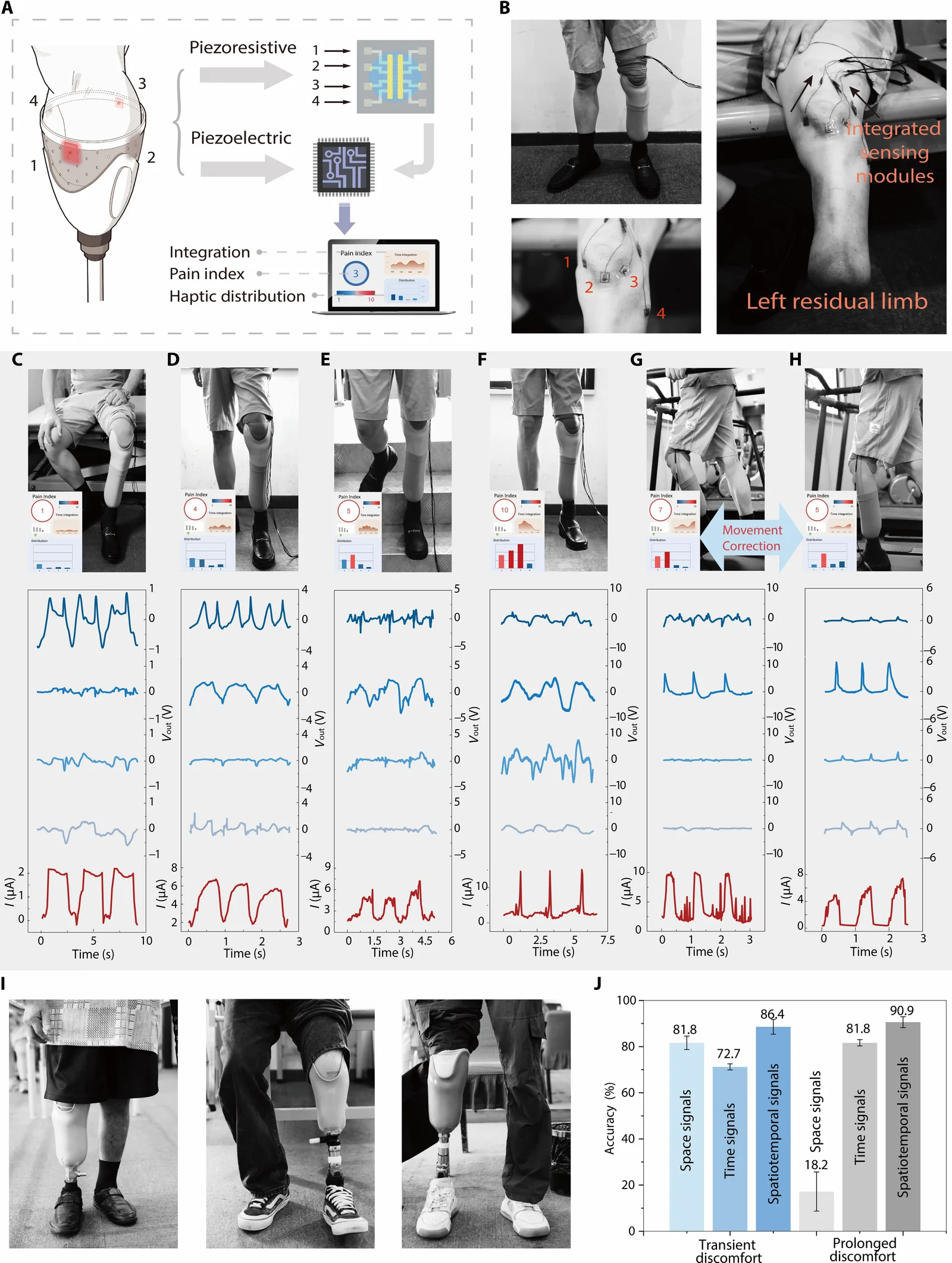
Feasibility trials with participants with transtibial amputation. (A) Schematic illustrations of the artificial perceptual system assisting participants with transtibial amputation in haptic localization and pain assessment. (B) The optical image of an amputee participant with the artificial perceptual system on the left residual limb. Representative haptic and pain data collected while a participant was (C) sitting, (D) walking, (E) climbing stairs, (F) jumping, and running before (G) and after (H) posture adjustment during prosthetic training. (I) The optical images of 3 participants with different amputation conditions. (J) Pain assessment of participants toward the feedback of the artificial perceptual system.

People who undergo lower-limb amputation face persistent challenges, including mobility impairments and PLP. Although prosthetic limbs are prescribed to enhance function and reduce fall risks, optimal rehabilitation outcomes rely on more than mechanical substitution. A critical factor affecting quality of life is the fit between the residual limb and the prosthetic socket, especially as limb volume fluctuates during recovery. Excessive or uneven pressure at the socket–skin interface can overload tissues, resulting in discomfort, inflammation, or ulceration. Thus, re-establishing somatosensory feedback—particularly haptic and pain input—is vital not only for evaluating prosthetic fit, but also for enabling protective responses to abnormal pressure. Nociceptive cues serve as early warnings to prevent potential tissue injury, while haptic distribution data help detect asymmetries in loading or gait, supporting posture training and biomechanical balance. We developed a real-time feedback system that dynamically integrates haptic distribution and pain assessment from the residual limb–socket interface to guide precise and voluntary movement adjustments (Fig. 6A). For prosthesis-residual limb interface assessment, the BPS system was deployed according to a patient-specific, anatomically guided sensing strategy. Four sensing sites were selected on the residual limb of a participant with transtibial amputation, targeting interface regions susceptible to excessive mechanical loading because of bony prominences or limited soft-tissue coverage (Fig. 6B). The final positions were further determined by the participant’s reported discomfort during daily prosthesis use, enabling localized monitoring of pressure-induced tactile and pain responses at clinically relevant contact regions.

Feasibility trials were conducted on participants with transtibial amputation, primarily to monitor movements such as sitting, walking, climbing stairs, jumping, and running during prosthetic training (Fig. 6C to H), with the pressure ranges of these activities falling within the sensor’s measurement range [71]. The real-time recordings of haptic and pain feedback indicate the pressure distribution and pain warning, providing effective methods for correcting poor training postures and accelerating rehabilitation. For instance, in the running condition, the increased knee flexion speed, greater femoral forward glide, and dynamic load variations may result in more increased pressure on the tibial tuberosity, requiring amputees to adapt to higher localized pressures. Haptic distribution and pain assessment revealed that excessive hip hiking, a common compensatory mechanism to assist prosthetic limb clearance, resulted in asymmetrical loading patterns and elevated socket pressure on the anterior tibial area. This compensatory hip movement may destabilize pelvic alignment and increase the risk of falls (Fig. 6G). Based on this feedback, participants were able to adjust their gait and reduce excessive pelvic motion, promoting safer and more biomechanically efficient running posture (Fig. 6H). Other participants, based on different amputation types, conducted feasibility trials and pain assessments to evaluate the effectiveness and comfort of the artificial perceptual sensor designs (Fig. S19 and Fig. 6I), ensuring that the wearable device could meet the specific needs and challenges associated with different levels of amputation. The discomfort levels of participants during the wearing and training period were evaluated by combining patient self-reports (Numerical Rating Scale, Table S3), experimenter observations (Faces Pain Scale—Revised, Table S4), and outputs from our artificial nervous system, demonstrating consistently high accuracy relative to the use of haptic spatial-only signals or pain temporal information, for both instantaneous and long-term discomfort (Fig. 6J).

## Discussion

The BPS introduced here adopted the mechanical synergistic design of the haptic and pain elements to create a neuromorphic perception system for spatiotemporal decoding haptic and pain information. The presented artificial device has resolved the conflict between the spatiotemporal localization of haptic perception and the spatiotemporal integration of pain perception, achieving an optimal trade-off between these 2 sensory modalities. Compared with existing artificial sensory systems based on pressure mapping or fixed-threshold warning, the artificial perceptual neuron provides more comprehensive haptic and pain-like feedback by emulating the coordinated mechanisms of human tactile and pain perception, including stimulus accumulation and injury sensitization (Table S5). The haptic and pain sensing feedback provided by this system endow the robotic manipulator with human-comparable behavior to accomplish challenging tasks of interacting with external stimuli in a safe and appropriate manner.

Furthermore, we demonstrate that the BPS enables participants with a transtibial amputation to evoke both haptic and pain sensations, facilitating neuroprosthetic adaptations to different locomotion modes. The current synergistic strategy for haptic and pain perception can be easily adapted to assess prosthetic fit, making it a promising intervention for restoring sensations and enhancing function in lower-limb amputees. Expanding the number of participants will enhance our understanding of the underlying mechanisms that contribute to haptic and pain sensing in individuals with prosthetic limbs. By including a broader diversity of amputation types (i.e., transfemoral, transtibial, and other variations), we can gain insights into how different residual limb conditions and their interactions with prosthetic interfaces affect sensory systems. We have further improved the compact and socket-integrated implementation of the BPS by integrating the sensing units into the prosthetic interface and optimizing the mounting structure (Fig. S20). Nevertheless, further efforts are still needed to improve wireless integration, medical-grade encapsulation, long-term wearing stability, and robust sensor fixation under dynamic gait conditions. We expect that these BPS will facilitate the development of more effective strategies and technologies for intelligent prosthetics, ultimately improving the quality of life for amputees.

## Ethical Approval

All experiments with human subjects were approved by the Human Research Ethics Committee of Zhejiang Sci-Tech University (Reference No. 202504E001). Informed consent was obtained from all the subjects.

## Data Availability

The data that support the plots within this paper and other findings of this study are available from the corresponding authors on reasonable request.

## Supplementary Materials

Supplementary 1 Texts S1 and S2 Figs. S1 to S20 Tables S1 to S5 Movies S1 to S4
