# Supplementary material for: A Bioinspired Perceptual Sensor for Spatiotemporal Decoding of Haptic and Pain Stimuli in Prostheses
Source: Cyborg Bionic Syst. 2026 Sep 22;7:0679. doi: 10.34133/cbsystems.0679 (PMC13594425; doi:10.34133/cbsystems.0679)
Supplement: Supplementary file 1 [file cbsystems.0679.f1.zip › Supplementary-materials.docx]

Supplementary Materials for

**Uniform and complete irreversible electroporation on gastric mucosa enabled by sintered silver hydrogel**

**This file includes:**

Supplementary Text

Fig. S1 to S33

Table S1 and S2

Legend for Movie S1 and S2

**Other Supplementary Materials for this manuscript include the following:**

Movie S1 and S2

Supplementary Note

Additional Materials and Methods

Simulation

The simulation model was 23 mm in length, 14 mm in width, and 5 mm in height. The central electrode has a length of 11 mm, a line width of 2 mm, and a terminal fillet radius of 1 mm. The peripheral ring electrode has a major axis of 20 mm, a minor axis of 10 mm, and a line width of 2 mm. The spacing between the ring electrode and the central electrode is also 2 mm. The model mesh was a free triangular mesh, and the mesh at the interface was refined to achieve higher computational accuracy in the model.

Swelling experiment

The hydrogel samples prepared for the swelling experiment were all the same size, 10 mm in length, 10mm in width, and 1 mm in thickness. The sintering solution and deionized (DI) water were both 5 ml in volume, which was sufficient to submerge the hydrogels. The initial weight of the hydrogel was noted, and it was then kept in a shaker at 37 ℃. The weights at the time points of 1, 6, 12, 24, and 48 hours were recorded and then divided by the initial weight to obtain the swelling ratio. Compared with soaking in DI water, the conductive hydrogel immersed in the sintering solution swelled more slowly. Moreover, we swelled the neat hydrogel in DI water. The swelling ratio of the neat hydrogel in DI water was much higher than that of the Ag hydrogel in sintering solution and DI water.

Biocompatibility experiment

*In vitro* experiment: normal gastric epithelial cells (GES-1) (SCSP-5471, National Collection of Authenticated Cell Cultures, China) were cultured in Dulbecco's Modified Eagle Medium (DMEM) (C3103, VivaCell, China), supplemented with 10% fetal bovine serum (C04001-050, VivaCell, China) and 1% penicillin-streptomycin (C3421, VivaCell, China). Every hydrogel sample (20 mm × 20 mm × 0.6 mm) was placed in 2 ml of complete medium and incubated at 37 ℃ for 24 hours to prepare the conditioned medium. Cells treated with pristine complete medium without hydrogel incubation and 75% ethanol are used as the negative control and positive control, respectively. GES-1 cells were seeded into 96-well plates and 6-well plates, cultured at 37 ℃ under 5% CO_2_ for 0.5, 1, 6, and 24 hours. Cell viability was assessed using Cell Counting Kit (M4839, AbMole Bioscience, USA) and Calcein/PI Live/Dead Viability/Cytotoxicity Assay Kit (C2015M, Beyotime, China), following the manufacturer’s instructions.

*In vivo* experiment: The hydrogels were cut into 10 mm in length, 10mm in width, and 1 mm in thickness. Nine rats were randomly divided into three groups: the Ag hydrogel group, the neat hydrogel group, and the sham operation group. The hydrogel samples were buried subcutaneously in the back of the rat. The sham operation group only underwent the surgical operation of incision and suture. Epidermis and subcutaneous tissues adjacent to the hydrogels were collected at 14-day endpoints. Samples were fixed in formalin fixative (BL539A, Biosharp, China), and cross-sectional samples were stained for histological analysis.

To evaluate the release of Ag ions, 4 vol% sintered Ag hydrogels (with a mass equivalent to that used in *in vivo* experiments) were placed in simulated gastric fluid and incubated at 37 ℃ for 10, 20, 30, 40, 50, and 60 minutes, as well as for 24 and 72 hours. Ag ion concentrations were measured using ICP-MS. To assess *in vivo* Ag deposition in distant organs and rule out systemic toxicity, sintered Ag hydrogels were administered into the stomachs of rats using a gavage needle; the control group received only the operation without the hydrogel. After 1 hour and 24 hours, the heart, liver, spleen, lungs, and kidneys were harvested for tissue ICP-MS analysis of Ag content and H&E staining, respectively.

Histology

Histological analysis was performed to characterize the changes in cellular morphology, extracellular matrix, and vascular structure within the target tissue area after IRE. Biopsies were taken from the stomach 1 hour after IRE. The biopsies were fixed in formalin fixative at 4 °C for 72 hours before transfer to 70% ethanol. Tissue samples were then embedded in paraffin and cut into 5 μm-thick tissue sections as previously described with minor modifications[10]. Sections that were located along the long axis of the positive electrode were selected for subsequent staining and histological analysis. H&E staining, TUNEL, and immunofluorescence were performed following the standard operating protocol. The antibodies used in fluorescence staining were TUNEL (G1504, Servicebio, China), anti-cleaved-Caspase-3 (GB115733, Servicebio, China), and CD31 (GB11063-2, Servicebio, China). The slides were scanned with a digital slide scanner (Pannoramic SCAN II, 3DHISTECH Ltd., Budapest, Hungary) and were analyzed with SlideViewer 2.6 (3DHISTECH Ltd., Budapest, Hungary). Histopathological analysis was performed independently by experienced pathologists with more than 5 years of experience.

In the group of metal electrode with a sintered Ag hydrogel interface, the fluorescence staining of TUNEL (green: TUNEL; blue: DAPI) and cleaved-Caspase-3 (red: Caspase-3; blue: DAPI) showed a homogeneous and substantial fluorescence signal, implying that the corresponding area of cell death after IRE. However, in the group of metal electrode with a neat hydrogel interface, the distribution of fluorescence signals of TUNEL and cleaved-Caspase-3 was very weak, indicating insufficient ablation. Moreover, in the group of metal electrodes without a hydrogel interface, the distribution of fluorescence signals was obviously inhomogeneous, which could reflect the unevenness of the electric field distribution. The differences in the distribution of fluorescent signals under the three aforementioned distinct interface conditions can fully reflect the critical role of conformal and effectively conductive bioelectronic-tissue interfaces in IRE. The CD31 (red: CD31; blue: DAPI) fluorescence signals showed the same trend in vascular response. In the group of bare metal electrodes with sintered Ag hydrogel interface, the vascular structure was well preserved, implying the safety of mucosal IRE (Fig. S28).

Supplementary Figures

Fig. S1. Simulation model. (A) The top view of the simulation model. The top of the model showed the clear shape of the circuit (purple) and grooves. (B) The side view of the simulation model. The depth and width of rugae on the tissue surface were both 1 mm.

Fig. S2. Meshing diagram of the multi-physics simulation.

Fig. S3. Conductivity Measurement. (A) A multimeter measured the resistance of a sintered Ag hydrogel strip sample (60 mm × 10 mm × 1 mm). (B) The sample was connected to Ag tapes through EGaIn, and the clamps connected the Ag tape to the multimeter.

Fig. S4. Schematic diagram of the assembly of the sintered Ag hydrogel interface and metal electrode.

Fig. S5. The schematic of the anchoring electrode using a pair of magnets.

Fig. S6. H&E staining of conformal contact between the mucosa and sintered Ag hydrogel. ( The color of the hydrogel has been adjusted to make the image clearer.)

Fig. S7. Steps for synthesizing the sintered Ag hydrogel. (A) PAAm-SA solution was the matrix solution; (B) Add Ca^2+^ to connect SA and enhance the hydrogel network. (C) Ag flakes were added to achieve high conductivity. (D) The sintering process made the Ag flakes connect tightly and form electronic conductive pathways. (Created in BioRender)

Fig. S8. The electrical resistance of Ag hydrogels with different Ag contents during partial dehydration. The resistance of the Ag hydrogel decreased over time. Ag hydrogels with Ag contents of 2 vol% and 4 vol% showed a significant decrease in resistance.

Fig. S9. Schematic diagram of Ag hydrogel before and after sintering. (A) Ag flakes were in a disordered state and scarcely connected before sintering. (B) Ag flakes were removed of surfactants and connected to form electronic conductive pathways after sintering. (Created in BioRender)

Fig. S10. The trend of swelling ratio changes of hydrogels soaked in sintering solution and deionized water for 48 hours. Red: Ag hydrogel in sintering solution; Blue: Ag hydrogel in DI water; Light Purple: Neat Hydrogel in DI water.

Fig. S11. The resistance changes of the sintered Ag hydrogel and the neat hydrogel during 50% cyclic stretching.

Fig. S12. Resistance of Ag hydrogels soaked in common solutions. The resistance changes were continuously recorded for 10 hours in each solution condition. At the fifth minute of recording, the solution was added to record the resistance changes of the Ag hydrogel during swelling.

**Fig. S13. Electrical conductivity of the hydrogel before and after ablation in an *ex vivo* porcine stomach model (pre-ablation: (1.77 ± 0.19) × 10^4^ S/m, post-ablation: (1.48 ± 0.26) × 10^4^ S/m, n = 5, *p*>0.05).**

Fig. S14. Live/Dead viability fluorescence images of the negative control, coincubated in sintered Ag hydrogel-soaked DMEM for 0.5 h,1 h, 6 h, 24 h and positive control groups. Green fluorescence indicates viable cells, while red fluorescence indicates dead cells.

Fig. S15. *In vivo* biocompatibility test. The H&E staining images of the subcutaneous tissues after sham surgery (control) or after placing the sintered Ag hydrogel and neat hydrogel subcutaneously in the back of the rats for 14 days.

Fig. S16. Ag ion release concentration from sintered Ag hydrogel when incubated in SGF water at 37 °C over a period of 10minutes, 20 minutes, 30 minutes, 40 minutes, 50 minutes, 60 minutes, 24hours and 72hours.

Fig. S17. ICP–MS analysis of Ag ions accumulated in heart, liver, spleen, lung and kidney after sham surgery (control) or placing the sintered Ag hydrogel in the stomachs of rats for 1 hour and 24 hours. (∗: *p* < 0.05; ∗∗: *p* < 0.01)

Fig. S18. The H&E staining images of the heart, liver, spleen, lung and kidney after sham surgery (control) or placing the sintered Ag hydrogel in the stomachs of rats for 1 hour and 24 hours.

Fig. S19. Adhesion performance of the sintered Ag hydrogel. (A) Schematic of the lap shear test (B) Shear strength curve of the sintered Ag hydrogel with porcine stomach (C) Shear strength curve of the sintered Ag hydrogel with metal electrode. (D) Statistical analysis of shear strength.

Fig. S20. Schematic diagram of an *in vitro* experiment on a potato cube. (Created in BioRender)

Fig. S21. The definition of the depth ratio on the potato cube model. *d_1_* was the deepest ablation depth in the central area, and *d_2_* was the ablation depth of the neighboring intrusive region center.

Fig. S22. Molds for potato IRE. (A) The tool for cutting potato slices into 30 mm × 20 mm ×10 mm cubes. (B) The mold for scribing grooves on potato cubes. (C) The 3D-printed resinous mold for grooves of 1 mm depth and 1 mm width. (D) The 3D-printed resinous mold for grooves of 1 mm depth and 2 mm width. (E) The 3D-printed resinous mold for grooves of 2 mm depth and 2 mm width.

Fig. S23. The depth ratio of the metal electrodes with sintered Ag hydrogel interface, metal electrodes with neat hydrogel interface, and bare metal electrode in the varied groove models. (∗: *p* < 0.05; ∗∗: *p* < 0.01)

Fig. S24. Endoscopic ablation of swine. The EACS was inserted into the stomach along with a gastroscope.

Fig. S25. The TPU supporting substrate of the electrode. (A) The TPU supporting substrate containing an embedded NdFeB magnet that works with external magnets to secure the electrodes. (B) The way to combine the magnetic-anchoring-assisted EACS with the endoscope is through the ring on the TPU supporting substrate.

Fig. S26. Magnet anchoring. (A) Schematic diagram of magnet anchoring (The external magnet created in BioRender). (B) The embedded NdFeB magnet in an EACS.

Fig. S27. The maximum ablation depth of the porcine stomach under different interface conditions.

Fig. S28. Immunofluorescence of gastric mucosal IRE under different bioelectronic-tissue interface conditions. (*From left to right: metal electrode with a sintered Ag hydrogel interface, metal electrode with a neat hydrogel interface, and bare metal electrode only*). (A) Representative TUNEL images. Scale bars, 500 μm. (B) Enlarged images from TUNEL. Scale bars, 100 μm. (C) Representative Cleaved-Caspase-3 images. Scale bars, 100 μm. (D) Representative CD31 images. Scale bars, 100 μm.

Fig. S29. High-magnification H&E stain of the submucosa and muscularis propria beneath the center of the ablation region. SM: submucosa; MP: muscularis propria.

Fig. S30. The Impedances between the mucosa and (A) metal electrodes with sintered Ag hydrogel interface, (B) metal electrodes with neat hydrogel interface, and (C) bare metal electrodes before (blue line) and after (red line) ablation, respectively.

Fig. S31. Sintered Ag hydrogel after ablation.

Fig. S32. SEM of sintered Ag hydrogel after ablation. (A) SEM of the section of the sintered Ag hydrogel after ablation. (B) SEM of the surface of the ablated sintered Ag hydrogel.

**Fig. S33. The ablation area of the ablation samples at the central cross-section of the rat liver models.**

Movie S1. The tensile test of the sintered Ag hydrogel.

Movie S2. Assembly of the electrode and the endoscope.

Table S1. Parameters of the simulation model materials

| Materials | Electrical conductivity  (S/m) | Thermal conductivity  (W/ (m·K)) | Density (kg/m³) | Relative permittivity |
| --- | --- | --- | --- | --- |
| Tissue | 0.333 | 0.52 | 1079 | 1 |
| Metal electrode | 2.22 × 10^6^ | 15 | 7900 | 1 |
| Conformal interface | 0.33~1000 | 25.62 | 1326 | 1 |
| Spacer | 0.01 | 25.62 | 1326 | 1 |
| Digestive juice | 0.8 | 0.6 | 1011 | 70 |

Table S2. Comparison of existing IRE-related electrodes

| IRE electrode | Balloon electrode [22][60] | Needle electrode [61-62] | Planar metal electrode [63-64] | PEDOT-coated electrode [65-66] | Sintered Ag hydrogel electrode |
| --- | --- | --- | --- | --- | --- |
| Material | NiTi/ Au | Stainless steel / Nitinol | Stainless steel / Pt / Au | Metal substrate + PEDOT: PSS coating | hydrogel |
| Conductivity | 10^5^~10^7^S/m | 10^6^~10^7^S/m | 10^6^~10^8^S/m | 10^3^~10^5^S/m | >10^4^ S/m |
| Young's modulus | 10^6^-10^8^ kPa | 10^8^-10^9^ kPa | 10^8^-10^9^ kPa | 10^3^-10^4^ kPa | 91 kPa |
| Contact | Stretchable Macro-scale contact | Invasive puncture | Rigid surface contact | Rigid planar microscale contact | Conformal (non-invasive) |
| Application sites | Cardiac | Solid tumors | Superficial tumors or surgically exposed tissue | Cell electroporation and transfection | Gastric |
